# Myriapods (Diplopoda and Chilopoda): medical aspects of envenomations

**DOI:** 10.1590/0037-8682-0104-2025

**Published:** 2025-06-16

**Authors:** Vidal Haddad, Ariadne Mendes Vidal Haddad, João Pedro Barreiros

**Affiliations:** 1 Universidade Estadual Paulista, Faculdade de Medicina de Botucatu, Departamento de Dermatologia e Infectologia, São Paulo, Brasil.; 2 Faculdade de Medicina de Catanduva, Centro Universitário Padre Albino - UNIFIPA, São Paulo, Brasil.; 3 Centre for Ecology, Evolution and Environmental Changes, Azorean Biodiversity Group, Global Change and Sustainability Institute, Lisbon, Portugal.; 4 Faculty of Agricultural Sciences and Environment, University of the Azores, Angra do Heroísmo, Portugal.

**Keywords:** Bites and Stings, Toxins, Myriapods (MeSH)

## Abstract

The Subphylum Myriapoda is part of the Phylum Arthropoda, and has two Classes related to human medicine. The Diplopoda Class comprises the Millipedes that poison through contact of their toxins with the skin and mucous membranes. The Chilopoda Class, the Centipedes are venomous animals that cause painful envenomation through stings. The clinical manifestations of human injuries caused by myriapods are typical. Because of the frequency with which these animals are found in domestic environments, it is important that this information be transmitted to medical teams and the general population.

## INTRODUCTION

The Subphylum Myriapoda is part of the Phylum Arthropoda, and has two Classes related to Human Medicine. The Diplopoda Class comprises the Millipedes, which are animals that poison their prey through contact with the skin and mucous membranes and ejection of toxins. The Chilopoda Class, the Centipede, are venomous animals that causes painful envenomation through stings^1^ ^,^ ^2^. Both Classes can be found in wild and domestic environments, and as the injuries most often do not have systemic repercussions, there are no statistical reports on the frequency of occurrence. However, due to the ease with which these animals are found inside homes, it is suspected that these negative interactions are common^1^ ^-^ ^3^. 

These animals have an elongated body shape and can be confused with each other for this reason; however, there are important differences in the morphology and envenomation capacity of each Class.

## DIPLOPODA (MILLIPEDES)

**FIGURE 1:**
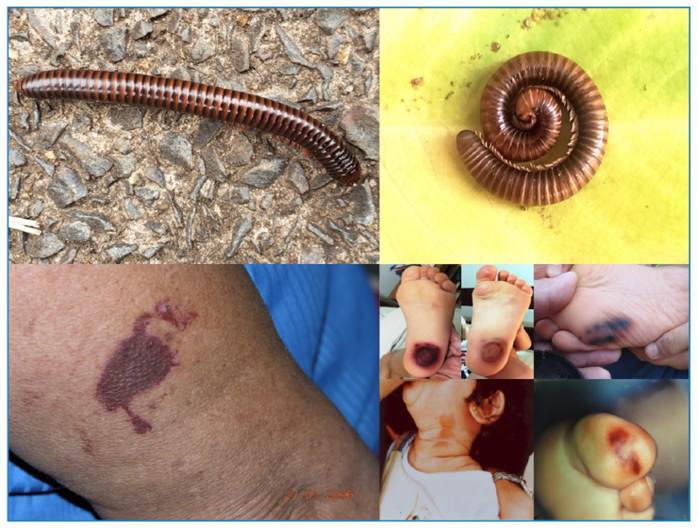
Top, Left: Diplopoda or Millipede present an elongated body and two pairs of legs in each segment. Top, right: The coiled position of a Millipede gives more resistance to its exoskeleton. Below: Millipede envenomation cause marked hyperchromic macules and confusion with other cutaneous and systemic diseases.

Diplopoda or Millipedes are cylindrical animals with a body divided into segments, from which two pairs of legs originate on each side, a distinctive factor of the Class (Figure 1). The cephalic segment has two antennae. There are many species of Millipedes that are widely distributed over the planet, especially in tropical and semi-tropical regions. They are slow animals that feed on plant debris, live in damp and poorly lit places, and are active at night^1^ ^-^ ^3^.

Because of their slowness, these animals have two defense mechanisms. The first is the coiling of the body, which takes on a rounded appearance, increasing the resistance of the exoskeleton (**Figure 1**). The second and most important is the discharge of foul-smelling fluids that irritate predators and human skin through pores on the sides of body segments^4^ ^,^ ^5^. 

These fluids are composed of alkaloids, benzoquinones, phenols, terpenoids, and hydrogen cyanide. These substances can repel predatory insects and cause caustic lesions on the skin and eyes of larger predators^4^ ^,^ ^5^. There have been reports of capuchin monkeys (*Cebus*spp.) and lemurs rubbing millipedes on their bodies to repel insects; some of these compounds have antifungal activity as well^6^ ^,^ ^7^. An accessory muscle is attached to these poisonous glands and pores, facilitating the expulsion of secretions.

**FIGURE 2:**
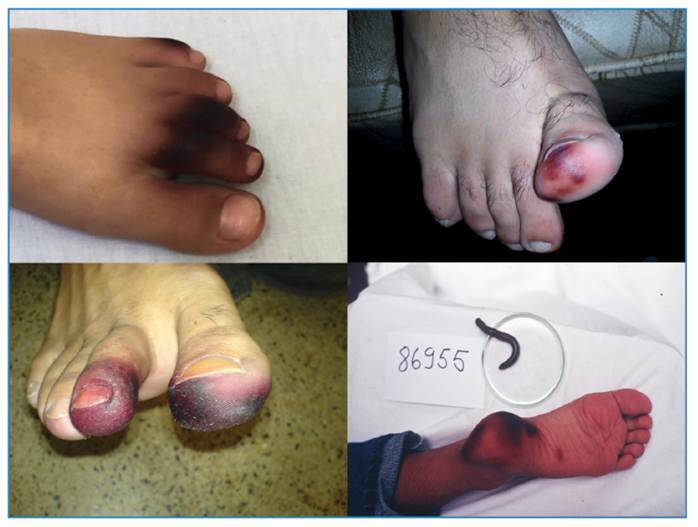
The similarity of Millepede envenomation with arterial obstructive diseases caused by atherosclerosis and *diabetes mellitus* causes stressful situations in emergency care.

Human poisoning most often occurs when millipedes penetrate shoes and other dark places in homes, and are compressed or crushed. Children are also victims of poisoning because of their curiosity. These fluids are released and interact with the human skin, initially causing an inflammatory condition with erythema, edema, and a burning sensation, which can be serious when it affects the eyes. Washing the skin with alcohol or ether (solvents) could be helpful immediately after contact. In this initial phase (one or two days), vesicles, blisters, and exulcerations may appear, and hyperchromic coloration may already be present^8^ ^-^ ^10^ (Figures 1 and 2). 

Subsequently, the location becomes hyperchromic, with colors varying between yellow, brown, and black at the point of contact with the animal, sometimes reproducing its body shape^9^ ^,^ ^10^ (Figures 1 and 2). This pigmentation can persist for months and does not require treatment, disappearing spontaneously. However, if the affected location is on the extremities (especially the lower limbs), it can lead to complex differential diagnoses (especially in the elderly and patients with diabetes ), as the colors are confused with manifestations of arterial occlusions and necrosis of the extremities (Figure 2). In this case, the pulses will be weak or absent, and the local temperature will be low, which does not occur in injuries caused by Millipedes.

## CHILOPODA (CENTIPEDES)

**FIGURE 3:**
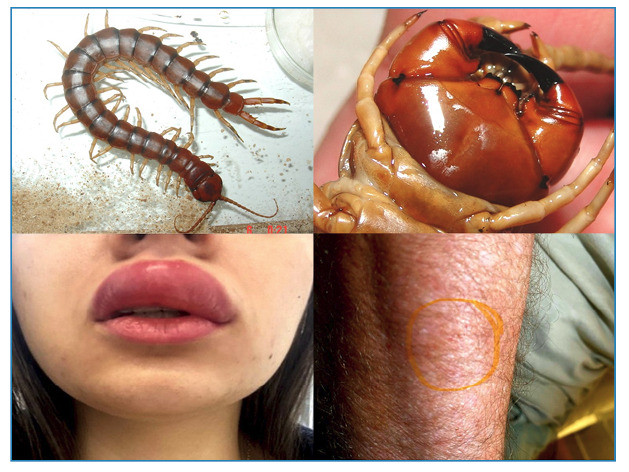
Top, left: Chilopoda or Centipede are venomous animals have a metameric body with only one pair of legs per segment. Top, right: The Centipede injects venom through forcipules, made up of the first pair of legs, which evolved into fang-like structures. Below: Centipede envenomation always cause intense pain and moderate local inflammation.

Chilopoda or Centipedes are animals with an elongated body shape divided into many segments (metameric). They can reach up to 30 cm in length. They are found in tropical and subtropical regions, and have a wide global distribution. Unlike Diplopoda, they have only one leg on each side of a segment, a differentiating factor between these animals^2^ ^,^ ^12^ (Figure 3).

Centipedes are agile invertebrates that make them fierce predators of other animals. Although they have a predilection for other invertebrates, especially cockroaches, there are reports of centipedes preying on snakes, amphibians, birds, and even mammals, such as bats^2^ ^,^ ^12^ ^,^ ^13^. 

They live in humid environments, under dry leaves and fallen tree barks and trunks. In domestic environments, where they are not rare, they can emerge from sewage pipes and drains, where they chase cockroaches^2^. 

These are venomous animals that inject toxins through forcipules or toxicognaths. The venom- inoculating apparatus of the centipedes comprises the first pair of legs, which have evolved into fang-like structures (Figure 3). These forcipules can reach a considerable size and inject large amounts of venom. The venom glands are located in the animal's head and run through the forcipules^14^.

The venom contains toxins with myotoxic and neurotoxic effects and a high percentage of inflammatory mediators, such as histamine and serotonin^14^. In humans, it causes local inflammation, bleeding, and intense pain at the point of the sting (Figure 3). Blisters and superficial necrosis of the skin are rarely observed. Although systemic effects are even rarer, low fever, malaise, and anxious states may occur, which are probably associated with pain caused by stings. Although not aggressive without provocation, centipedes can sting repeatedly, increasing the likelihood of intensely painful processes in victims. The possibility of allergic phenomena caused by the venom is real, but rare^11^ ^,^ ^15^ ^,^ ^16^. 

The benignity of the clinical manifestations seems to contribute to the undernotification of injuries, as victims do not always seek medical assistance. The causative agents in 136 cases (63%) were identified as belonging to the genera *Cryptops* (n=79), *Otostigmus* (n=45), *Scolopendra* (n=5), or others (n=7). Of the patients bitten by the *Scolopendra* genus, only 4% presented with erythema and 10% with edema. Only patients bitten by *Scolopendra* and *Otostigmus* required therapeutic treatment. The Scolopendridae family occasionally cause extensive dermonecrosis^17^ ^,^ ^18^ ^,^ ^19^ ^,^ ^20^. 

Centipede venom contains cytotoxins, proteases, neurotoxins and allergens^14^ ^,^ ^21^. Although fatalities are rare and not always proven, there are some trustworthy reports of deaths following centipede stings. In the USA, seven human fatalities due to centipedes were reported between 1991 and 2007; however, no cause was presented^22^ ^,^ ^23^. The most documented and reliable report occurred in a 7-year-old boy in the Philippines, who was stung in the cephalic segment and died approximately a day later^24^. Some deaths were reported by the press, but without scientific publications, such as a 21-year-old woman bitten in Thailand and a patient in Mauritius who accidentally ingested a centipede and died of suffocation following a sting in the throat. Systemic complications that should be monitored can be observed in the muscles, kidneys, and cardiovascular system, including myocardial infarction^25^ ^-^ ^30^. Centipedes have also been reported to feed on human corpses and can cause infections as serious as necrotizing fasciitis after stings^31^ ^,^ ^32^. Although rare, the possibility of allergic reactions is real^33^. 

The stings of these animals do not appear to be fatal, although they cause significant pain and stress to victims. Envenomation resolves spontaneously, but can be hastened by the use of analgesics, cold compresses, and antibiotics if there are signs of secondary infection^34^.

## CONCLUSIONS

The clinical manifestations of human injuries caused by Myriapods are typical. Because of the frequency with which these animals are found in domestic environments, it is important that this information is transmitted to medical teams and the general population. Envenomation caused by Millipedes may mimic vascular emergencies and should be recognized by physicians as harmless. The consequences of centipede stings can be serious, requiring emergency care.
